# Analytical challenges and opportunities in the extraction, separation, and identification of food phospholipids

**DOI:** 10.3389/fnut.2026.1743161

**Published:** 2026-02-17

**Authors:** Abir Bahi, Yazan Ranneh, Moza Saif Obaid Alyammahi, Mariam Faleh Hamad Alnuaimi, Carine Platat, Abdulmannan Fadel

**Affiliations:** 1 United Arab Emirates University, Department of Nutrition and Health, College of Medicine and Health Sciences (CMHS), Al-Ain, United Arab Emirates; 2 Al Ain University, Department of Nutrition and Dietetics, College of Pharmacy, Al Ain, United Arab Emirates

**Keywords:** extraction, food matrix, identification, methodological standardization, phospholipids, separation

## Abstract

Phospholipids (PLs) are minor but functionally important food lipids whose amphiphilic structure and strong interactions with the complex food matrix make their extraction and analysis challenging. This review summarizes a matrix-aware synthesis of current strategies for PLs extraction, purification, separation, and identification across major food sources, including eggs, dairy products, plant oils, marine oils, and microbial oils. While traditional solvent-based systems are the “gold standard,” new, emerging “greener” technologies such as supercritical CO_2_, ultrasound, microwave, enzyme-assisted, pressurized liquid extraction, and deep eutectic solvents show promise in terms of selectivity, efficiency, molecular integrity, and environmental sustainability. Due to the lack of universal standard methodology, this review emphasizes a tiered, objective-driven analytical framework in which extraction is adapted to the matrix origin, followed by cartridge solid-phase extraction for PLs enrichment, and progressively deeper analytical resolution. The most effective analytical approach combines orthogonal tools, including TLC for qualitative screening, UHPLC coupled with universal detectors (CAD/ELSD) for robust class-level quantification, LC–MS(/MS) for molecular-species identification and oxidation/lysophospholipid monitoring, ^31^P NMR for independent class-level validation, and GC-FAME for fatty-acyl profiling. The substantial variability among published results is attributed to non-standardized extraction methods, different detector responses, and matrix-dependent effects. Accordingly, a flexible, matrix-aware analytical workflow is proposed highlighting future priorities in standardization, automation, and validation of greener technologies and analytical strategies to achieve reproducible, comparable, and sustainable PLs analytical and production processes.

## Graphical abstract

**Figure:**
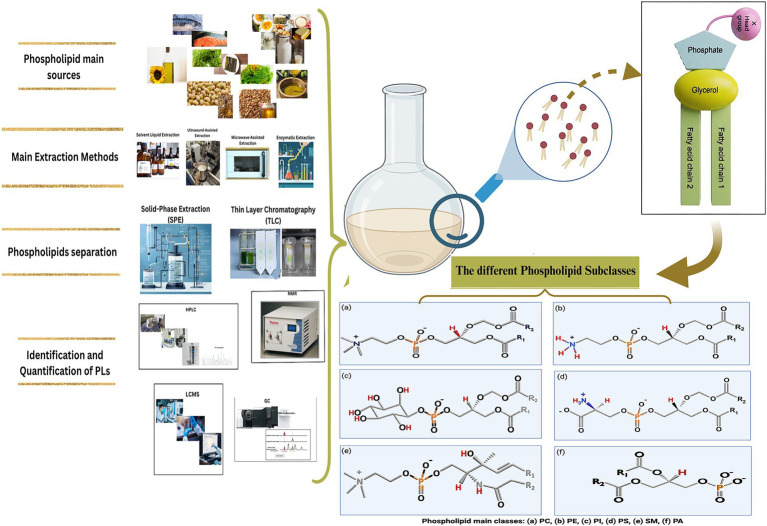
Main phospholipid sources and their main extraction, separation, and identification methods.

## 1 Introduction

Phospholipids (PLs) are unique lipids that play a crucial role in food systems due to their amphiphilic nature, contributing to emulsion formation, structural stability, and texture modulation in a wide range of products (1–3). Dietary PLs play a particular role as essential components of cellular membranes and lipid metabolism (4), as well as other emerging health-promoting functionalities. They contribute to the intake of choline and essential fatty acids and have been associated with beneficial effects on brain, liver, and cardiovascular health (5).

In fact, increasing evidence indicates that dietary PLs support brain health and cognitive function, as they are integral components of neuronal membranes and contribute to synaptic plasticity, neurotransmission, and age-related cognitive maintenance (6–8). Specific PLs classes, particularly phosphatidylserine (PS), phosphatidylcholine (PC), and plasmalogens (PL), and marine-derived PLs-bound ω-3 fatty acids, have been associated with neuroprotective effects, including reported improvements in memory, learning capacity, and executive function (9–11).

Moreover, PLs and their metabolites, particularly lysophospholipids, were recognized as key modulators of inflammatory and immune system response (12). They act as bioactive lipid mediators that influence immune homeostasis and the inflammatory process with implications for chronic inflammatory conditions and immune-related disorders (12–15). Specific PLs classes, such as Plasmalogens and phosphatidylserine, further contribute to antioxidant and anti-inflammatory protection by enhancing cellular resilience against oxidative stress, as extensively discussed in recent reviews (10, 16–18). From a technological perspective, PLs are widely used in food and nutraceutical formulations as natural emulsifiers, stabilizers, and delivery systems for lipophilic bioactives, due to their technological roles (1, 19, 20). These various physiological and technological functions are directly related to the dietary sources of PLs, which vary significantly in their composition and structural features.

The main food sources of PLs include eggs, dairy products, soybeans, fish, and oilseeds, which represent rich dietary sources of these compounds (5). This source diversity directly influences the functional properties of the PLs and their extraction methods (1, 20). This diversity is primarily controlled by three main factors, which are: fatty acid composition (length and saturation level), head group structure, which determines its charge and hydrophobicity (choline, serine, inositol …), and extraction approaches (21, 22). The variation in these factors affects membrane fluidity, emulsifying capacity, and oxidative stability (23). For instance, the ability of PLs as an emulsifier, which allows them to coat the oil droplets, is highly correlated with different factors such as the origin (source) of the PLs, the proportion of PC to other PLs, as well as the saturation of fatty acids (1). PLs from peony seeds, rich in PC (78.49%) and lysophosphatidylcholine (LPC) (8.11%), along with a considerable proportion of polyunsaturated fatty acids (PUFA: 88.84%), have an effective emulsification ability and produce uniform oil droplets (20). Meanwhile, soybean PLs, the most common commercial source of PLs with high content of PC, phosphatidylethanolamine (PE), phosphatidylinositol (PI), phosphatidic acid (PA), and high PUFA, have a strong ability to stabilize the emulsified system, mainly through protein complexes (23). They are tightly associated with neutral lipids, proteins, and carbohydrates, making degumming followed by solvent partitioning or supercritical CO_2_ de-oiling necessary to disrupt the lipid-protein interactions and selectively enrich PLs (24, 25). In contrast, egg yolk PLs, also frequently analyzed PLs type embedded within complex lipoprotein assemblies and membranes, contain a high percentage of PC (~72%), followed by PE, PI, PL, and sphingomyelin (SM) (26). There is also a diverse fatty acid profile that includes long-chain PUFA such as docosahexaenoic acid (DHA) and eicosapentaenoic (EPA) (10). The strong association of PLs with proteins and cholesterol in egg yolk necessitates solvent systems or emerging enzymatic and membrane-based approaches to enhance extraction efficiency while preserving PLs integrity (27, 28). Meanwhile, marine PLs are also valuable sources with a distinctive fatty acid composition that is particularly rich in omega-3 long-chain PUFA such as EPA and DHA. Methods to process and extract PLs from marine sources are more specialized and require milder conditions to safeguard their oxidative stability and biological activity (29–31). The significant source-dependent structural and compositional diversity directly complicates the extraction, separation, and analytical characterization of PLs across diverse dietary matrices.

One of the key challenges in PLs research is the lack of standardization of analysis procedures across different food matrices, which limits progress compared to neutral lipid studies. Besides their limited amounts and source-dependent variability, PLs analysis efficiency and reliability are additionally challenged by food matrix complexity and processing methods, which directly impact their extraction as well as their characterization (13, 32, 33). Components such as proteins, neutral lipids, polysaccharides, and pigments can strongly interact with PLs, lowering extraction efficiency and increasing the co-extracting interfering compounds, as seen in protein-rich foods like dairy and egg yolk (26, 34) or pigment-rich plant materials (35). Consequently, extraction strategies and cleanup methods often need to be adapted to the specific food matrix and the intended application to obtain reliable PLs data (36–38). These issues are further impaired by the lack of standardized analytical procedures across studies.

PLs extraction methods range from conventional solvent-based procedures (39, 40) to supercritical CO₂, enzyme-assisted, or ultrasound-assisted extractions (17, 18, 30, 31, 41–43). These methodologies limit the comparability of results across studies. Moreover, chromatographic characterization of PLs also lacks consistency with methods ranging from HPLC–MS to RP-HPLC–MS/MS, in addition to the limited consensus with key isomer distinctions (30, 42, 44–46). Furthermore, other challenges arise in quantifying PLs due to the broad reference standards and calibration used, which add a further layer of analytical inconsistencies in the current literature. However, updated methodologies have emerged that can address these traceability concerns, including HILIC-ESI-AIF-MS coupled with ^31^P NMR or ICP-MS (5, 44, 47–49). Yet, in a recent study on milk PLs, it has been reported that regardless of the use of advanced LC–MS systems for detection, differences in extraction and MS conditions are still altering the result substantially (50). These analytical variations restrict the reproducibility of research findings and hinder broader scientific and industry development.

There is a lack of consensus on the extraction, separation, and detection methodologies, which leads to results that are generally non-comparable with low quality, negatively affecting database creation, new product development, and process optimization. Our review comprehensively provides a synthesis and critical evaluation of existing extraction, separation, purification, identification, and quantification methods for PLs from different foods as well as other biological matrices.

## 2 Phospholipid classes and structures

**Figure 1:**
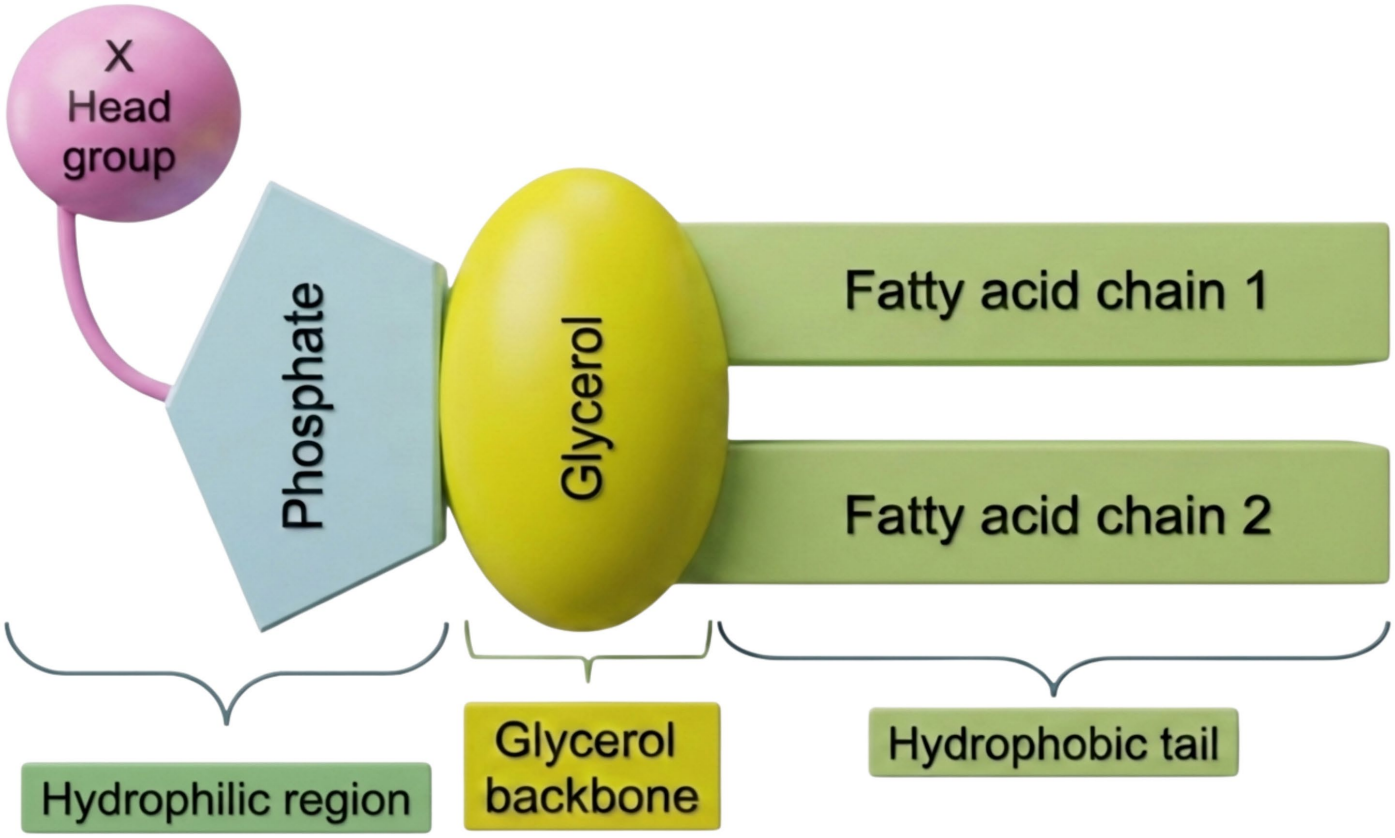
Generalized schematic architecture of glycerophospholipids. Backbone: Glycerol; two fatty acid chains (R1 & R2): hydrophobic tails; phosphate group (red): polar linkage site; head group (X): varies depending on the class.

Structure-wise, PLs are amphiphilic molecules, consisting of two hydrophobic fatty acid tails and a phosphate-based polar head group (51). Accordingly, PLs can be divided into subclasses based on their headgroups (Figure 1). The primary food glycerophospholipids are phosphatidylcholine, phosphatidylethanolamine, phosphatidylinositol, phosphatidylserine, phosphatidylglycerol (PG), and phosphatidic acid. All glycerophospholipids share a phosphatidic acid backbone, which includes a glycerol backbone with two fatty acyl esters and a phosphate group, with a phosphodiester-linked headgroup to choline, ethanolamine, inositol, serine, or glycerol (Figure 1) (11, 27).

**Figure 2:**
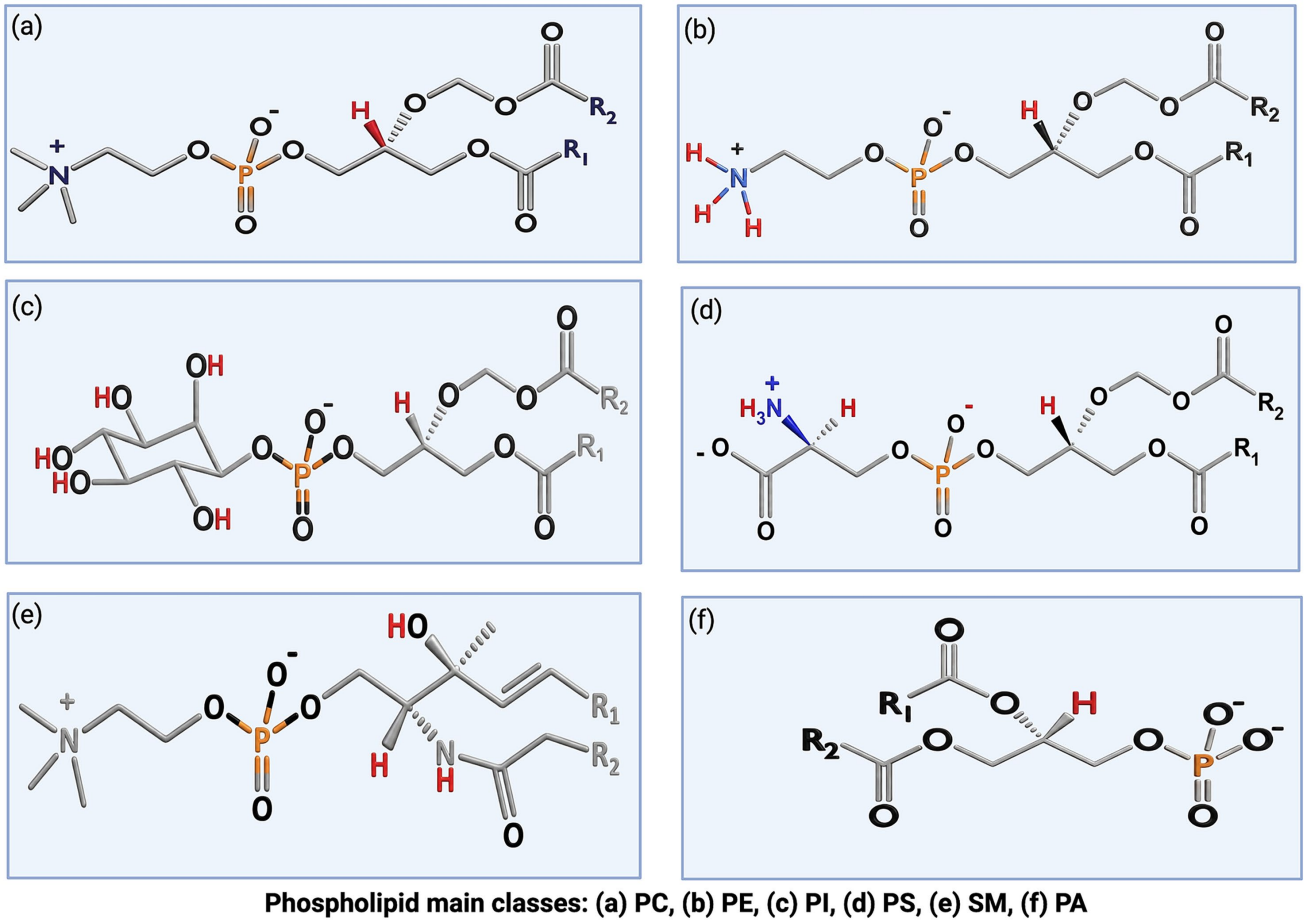
Representative canonical chemical structure of major phospholipid classes: **(a)** phosphatidylcholine (PC), **(b)** phosphatidylethanolamine (PE), **(c)** phosphatidylinositol (PI), **(d)** phosphatidylserine (PS), **(e)** sphingomyelin (SM), and **(f)** phosphatidic acid (PA). R₁ and R₂ denote variable fatty acyl chains.

PC is zwitterionic (bearing both positive and negative charges yet net neutral at physiological pH) and is generally the most abundant in egg yolk and muscle tissues (Figure 2) (41). Moreover, PE is also zwitterionic and may match PC. On the other hand, PI and PS are anionic at physiological pH and are often less abundant, although PS is found at ~2–11% of bovine milk PLs (27). PG is also anionic and rich in plants and microbes; its dimer, cardiolipin (CL), is abundant in heart tissue but found only in trace amounts in foods like beef (44, 52). Sphingophospholipids, predominantly sphingomyelin (SM), are also found in foods (Figure 2). SM has a ceramide backbone that includes sphingosine with a fatty acid and a phosphocholine headgroup (mirroring PC) and is highly abundant in milk and egg (27). Lysophospholipids-LPC and LPE are mono-acyl derivatives of PC and PE that can be formed during digestion or food processing. Though they generally existed at lower concentrations, these Lysophospholipids still need to be accounted for.

**Figure 3:**
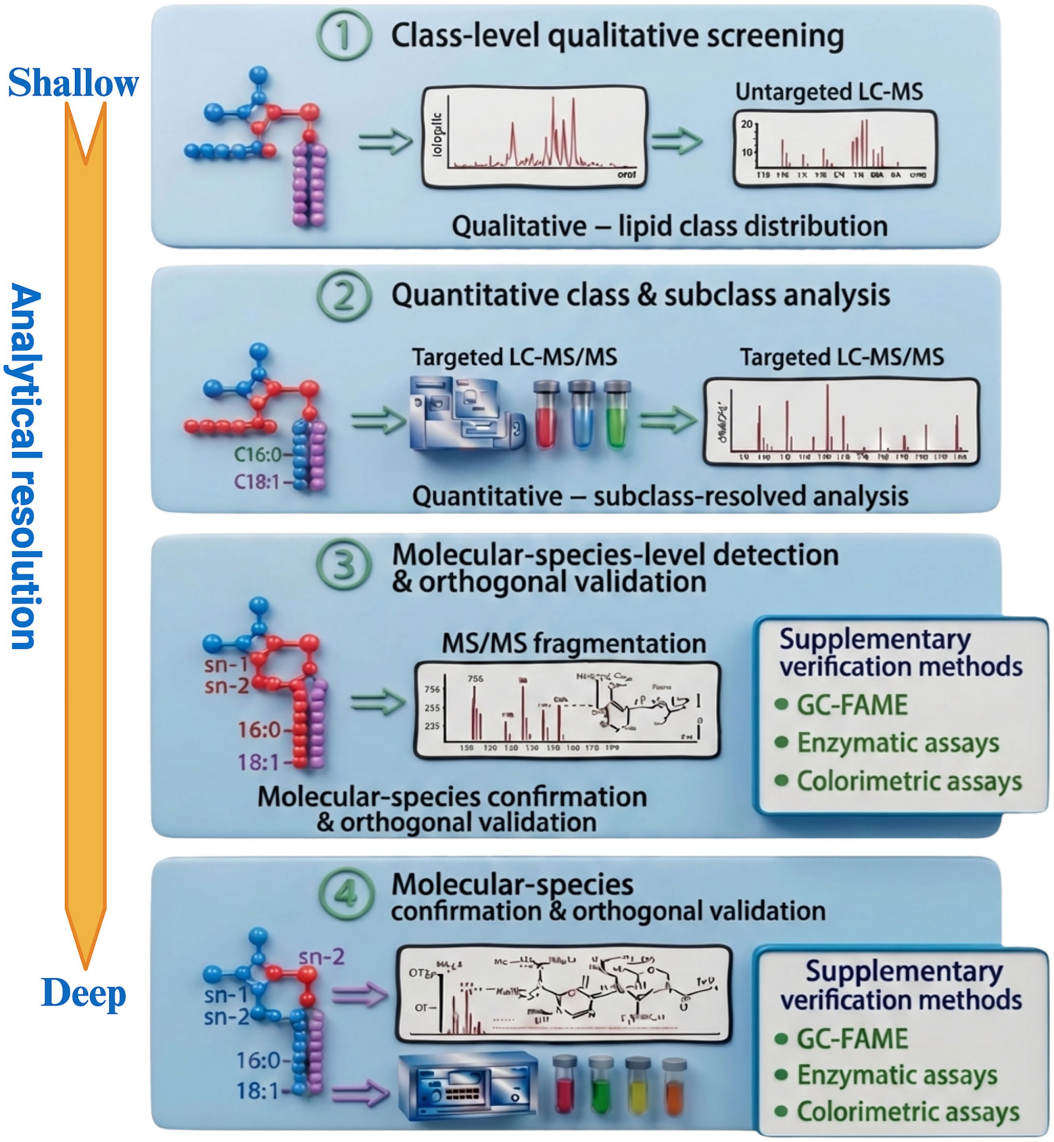
Analytical framework for phospholipid characterization from class-level screening to molecular-species-level confirmation (created in BioRender. Bahi, A. (2026) https://BioRender.com/ksl84qs).

Due to the amphiphilic nature, PLs usually assemble into membranes, micelles, and liposome-like structures and exhibit strong interactions with proteins, carbohydrates, and other food matrix components (27). These interactions create challenging conditions for their release from food matrices compared to neutral lipids (53–55). Moreover, most glycerophospholipids share a common phosphatidyl backbone and differ only slightly in head-group chemistry or fatty-acyl composition, resulting in extensive isobaric and isomeric overlap (56). This high degree of structural similarity among PLs classes represents a fundamental obstacle to their selective isolation and accurate characterization (Figure 2) (3). The highly variable fatty acyl composition also contributes to this complexity; for example, marine PLs are rich in omega-3 species while dairy and meat PLs are dominated by saturated and monounsaturated species (10, 54, 57). Additional variations in acyl-chain length, unsaturation, and positional isomerism further complicate the molecular identification of PLs, while quantitative analysis may be influenced by class-dependent ionization behavior and matrix-induced effects (55, 58). PLs classes also differ in charge properties, where PC and SM are neutral zwitterions, whereas PI, PS, PG, PA, and CL are anionic. These charge and polarity differences define PLs’ solubility and chromatographic behavior and directly affect the extraction and analysis process (29, 59, 60). As a result, this complex structural organization of PLs typically requires the use of mixed solvent systems capable of disrupting both polar and nonpolar interactions (29, 59). However, such approaches often promote co-extraction of other polar constituents, increasing sample complexity and contributing to incomplete recovery, particularly for low-abundance classes such as PI (61). These structure-driven limitations should be considered when analyzing phospholipid compositional data and provide the context for the analytical approaches discussed in section 4 (Figure 3).

## 3 Occurrence of phospholipids in food sources

Phospholipids are naturally present in foods of animal, plant, and microbial origin, and are common in eggs, milk, meats, fish and shellfish, grains, and oilseeds (Table 1). The most common sources of phospholipids in nature are egg yolk (10.3 g/100 g), beef brain (5.4 g/100 g), and animal livers (2.5–2.9 g/100 g) (8). The phospholipid composition in foods of animal, plant, and microbial origin is variable, as a consequence of the different structures and biological functions of these molecules (29, 60).

**Table 1:** Comparative distribution of key phospholipid classes across different food matrices.

Food sources	Major PL classes (%)						Reference
	PC	PE	PS	PI	SM	Other PLs	
Bovine milk (dairy)	12–48	3–39	2–16.1	–	4.1–29.2	–	Huang et al. (145) and Sun et al. (49)
Human milk	19–38	10–36	–	–	29–45.5	Minor PLs in decreasing order	Sun et al. (49)
Camel milk	27.0	40.5	4.1	4.1	24.1	–	Bakry et al. (144)
Buttermilk (Buffalo)	25.5	34.0	13.5	6.1	20.9	–	Di Paolo et al. (32)
Egg yolk	~71.0	~17.1	–	–	~2.1	LPC, LPE, Cer	Cheng et al. (10)
Soybean lecithin (de-oiled)	~23.0	~20.0	~1.0	~14.0	–	–	Wang et al. (150) and Zaaboul et al. (151)
Soy lecithin (liquid)	19–21	8–20	–	20–21	–	Other PLs (5–11)	List (147) and Robert et al. (22)
Atlantic salmon frame bone	79.6	9.7	2.8	7.9	–	–	Haq et al. (29) and Haq and Chun (68)
Anchovy	68.0	29.0		1.0		2.0	Haq and Chun (68)
Mitochondria	35–45	30–40	2–5	3–7	0	CL: 10–20	Kuschner et al. (74) and Kim and Hoppel (76)
S. aureus^*^	–	–	–	–	–	PG: 50–84%, L–PG: 10–30%, DAG: 3–5% (in some variant), CL: 1–7%	Hilton et al. (2), Jones et al. (73) and Jin et al. (78)
B. subtilis^*^	–	17–70	–	–	–	PG: 30–75%, L–PG: 2–3%, CL: 4–10%	
E. coli^*^	–	75	–	–	–	PG: 20%, CL: 5%	
*Yeast (S. cerevisiae)*	–	15–20	15–25	3–7	15–20	–	

^*^Microbial lipid profiles are dominated by PG, PE, and cardiolipin rather than PC and SM.

PC, Phosphatidylcholine; PE, Phosphatidylethanolamine; PI, Phosphatidylinositol; PS, Phosphatidylserine; PA, Phosphatidic acid; SM, Sphingomyelin; PG, Phosphatidylglycerol; LPC, Lysophosphatidylcholine; LPE, Lysophosphatidylethanolamine; L-PG, Lysophosphatidylglycerol; Cer, Ceramide; DAG, Diacylglycerol; CL, Cardiolipin.

Egg yolk is one of the richest natural sources of phospholipids, accounting for approximately 10% of its wet weight and nearly 30% of total yolk lipids. Its PLs composition is characterized by a strong predominance of PD, which typically constitutes more than 70% of total PLs, followed by PE at 15–24%. Minor classes, including LPC, LPE, and SM, together account for only a small fraction of total PLs pool. Owing to this compositional profile, egg yolk is widely recognized as an exceptionally rich source of choline-containing PLs (10, 62, 63).

Milk and dairy products have also been of interest as alternative commercial sources of phospholipids, particularly due to their richness in SM and PS (59). In bovine milk, the PLs fraction is dominated by PE and PC, with substantial contributions from SM, PS, and PI. In human milk, PLs represent a small proportion of total lipids (0.26–0.80%), however, SM is typically the most abundant phospholipid (29–45%) often exceeding PC, with a critical role in supporting infant neurodevelopment and gastrointestinal barrier function (49, 64). The second and third most abundant classes are PC and PE. The reported values for milk phospholipid composition can vary significantly, probably due to methodological differences in lipid extraction and analysis as well as biological factors like breeding period, diet, lactation stage, and technological influences such as processing conditions (28, 65, 66).

Natural phospholipids are extracted primarily from plant-derived and obtained from vegetable sources such as oilseeds (soybeans, sunflower, rapeseed), cereals, wheat germ, flaxseeds, oil-rich nuts, and some legumes Most plant tissues contain only minor amounts of PLs; however, seeds and beans represent the principal reservoirs due to their role in lipid storage (20). Crude vegetable oils contain low but significant PLs content (1–3%), which are further refined to obtain the purified lecithin (25). The dominant PLs classes, across plant-derived materials, are PC, PE, PI, and PS, with PC generally representing the major component of de-oiled vegetable lecithin. Minor constituents such as PA and Lysophospholipids, particularly LPC, are also consistently detected but at much lower levels. The primary and dominant commercial source of PLs is soybean oil (1.5–3% PLs), which yields lecithin with the highest total PLs content (65–75% PLs). PLs composition of plant lecithin is strongly influenced by agronomic variables, genetic background of the crop, environmental conditions, and processing parameters such as extraction and refining. These factors collectively account for the substantial variability reported in PLs subclass distribution across different plant sources and commercial lecithin products (13, 22, 67).

Marine sources, including finfish, shellfish, roe, and algae, represent an important dietary source of PLs, particularly distinguished by their enrichment in long-chain omega-3 polyunsaturated fatty acids such as EPA and DHA (27). Compared with terrestrial PLs, marine-derived PLs typically exhibit a higher degree of unsaturation, with omega-3 PUFAs preferentially esterified at the sn-2 position of the glycerol backbone. The total PLs content of marine organisms varies widely among species and tissues, reflecting differences in physiology and lipid storage. PC is the major class of phospholipid present across marine foods (45–80%), while PE, PI, and PS occur at lower but variable proportions. SM is generally present at relatively low levels in marine species compared with terrestrial animals, although elevated concentrations have been reported in certain shellfish. Notable exceptions to the typical class distribution are observed in specialized tissues such as fish roe, where PI can represent a major PLs class, underscoring its functional importance in reproductive and developmental processes (68, 69). In addition to class composition, marine PLs are characterized by a high degree of fatty acyl unsaturation. PC commonly contains substantial proportions of PUFAs, while DHA is frequently enriched in PE and PS, contributing to the distinctive nutritional and functional properties of marine PLs (29, 70).

Owing to their rapid growth, scalability, and controlled cultivation, microbial systems are increasingly explored as sustainable alternative sources of tailored PLs mixtures with distinct functional properties (71). Microbial sources of phospholipids, such as yeasts, fungi, and bacteria, are characterized by membrane compositions that differ markedly from those of higher plants and animals. Their phospholipid profiles are typically enriched in PE, PI, and CL, reflecting their central roles in maintaining membrane integrity, supporting bioenergetic processes, and enabling adaptation to environmental stress (72, 73).

Mitochondria are distinguished by their cardiolipin-rich membrane anatomy, where CL, PC, and PE comprise the core phospholipid framework (74). CL consistently represents a substantial fraction of the mitochondrial phospholipid pool, highlighting its conserved structural and functional significance in energy metabolism and membrane organization. The relative abundance of these PLs varies across tissues and physiological states (75, 76).

The extraction of food-grade PLs is directly influenced by the noted variability in the origin of each source, class distribution, fatty acyl composition, abundance, and matrix association, as well as the multi-step analysis processes involving concentration, solvent extraction, distillation, and fractionation steps which often result in complex workflows and low yields (41, 51). Plant-derived PLs are typically extracted from oil-rich plants by degumming or solvent fractionation approaches, whereas egg and dairy PLs, which are tightly associated with proteins and membranes, require more complex or multi-step extraction procedures (37, 38, 43, 60, 77). Marine-derived phospholipids are typically high in omega-3 PUFAs, rendering them highly susceptible to oxidative degradation during processing and analysis (29, 70). In contrast, PLs from cereal- and microbe-derived matrices are embedded in polar, structurally complex matrices, where solvent polarity and enzymatic accessibility strongly affect recovery (45, 60, 78). As a conclusion, these source-dependent structural and matrix-specific properties control PLs stability, extraction efficiency, and class selectivity, highlighting the necessity of customized analytical techniques for the PLs sources (29, 35). The implications of these differences for extraction strategy selection and downstream lipidomic analysis are discussed in Section 4.

## 4 Extraction of phospholipids from foods and raw materials

Recent advances in extraction technologies include both solvent-based and green methods, such as supercritical fluid extraction, ultrasound- or microwave-assisted extraction, and enzyme-assisted extraction (28). The continued development of improved extraction methods is still needed to increase the yield, purity, and eco-friendliness of phospholipid isolation (37, 38, 79). Variations in the lipid classes extracted and reported for food samples can still be observed, and no standard protocol is used for phospholipid extraction and quantitation in foods (37, 38, 54, 79).

### 4.1 Conventional and modified solvent-based extraction methods essays

Solvent extraction is the most common method for extracting lipids from food or biological matrices. The choice of solvents depends on the “like dissolves like” principle: a nonpolar solvent, such as hexane, can be used to extract neutral lipids, whereas methanol or ethanol will dissolve polar phospholipids (54, 80). In practice, the sample is mixed with the solvent, allowing the lipids to be extracted into the solvent phase. The lipids are then separated by washing or purification steps, while other substances are left behind (61).

#### 4.1.1 Conventional organic solvent systems

Folch (chloroform-methanol, 2:1) and Bligh and Dyer (methanol-water-chloroform) methods are considered the benchmark protocols for total lipid and phospholipid extraction to date (Table 2) (39, 40). The Folch method gives the best recovery from lipid-rich matrices like dairy or egg yolk, whereas Bligh and Dyer is more effective in low-fat or high-moisture matrices, such as tissue samples or milk (35, 81). Both can fully recover the major phospholipid classes but are less effective in minor acidic phospholipids (PA, PI, PS) that often remain in the aqueous layer using the Bligh and Dyer method (82). The main drawback of these protocols is the use of highly toxic and environmentally hazardous chloroform.

**Table 2:** Conventional and emerging solvent-based phospholipid extraction methods commonly used in food matrices: principles, advantages, limitations, and suitability.

Method	Principle	Advantages	Limitations	Suitability for food matrices	Reference
Folch	Chloroform-methanol (2:1 v/v) biphasic extraction; polar lipids partition into methanol–water, non-polar in chloroform	High recovery of total lipids and phospholipids; well-established	Uses toxic chloroform; bottom organic layer complicates handling; not food-grade	Suitable for high-fat tissues (egg yolk, brain, liver)	Folch et al. (40) and Ulmer et al. (148)
Bligh and Dyer	Chloroform-methanol–water (1:2:0.8 v/v/v) biphasic; optimized for low-fat samples	Efficient for dilute/low-fat samples; widely cited	Chloroform use; slightly lower yield for lipid-rich samples	Low-fat matrices like milk, serum, plasma	Bligh and Dyer (39) and Ulmer et al. (148)
Hara and Radin	Hexane-isopropanol (3:2 v/v) single-step extraction	Safer than chloroform; simple handling; good for neutral lipids	Under-recovery of highly polar lipids (phospholipids, gangliosides)	Neutral lipid-rich samples (egg yolk, oils)	Schoeny et al. (48) and Hara and Radin (85)
MTBE (Matyash)	MTBE-methanol–water biphasic; lipids in upper organic layer	Less toxic than chloroform; upper layer simplifies handling; good polarity coverage	Very polar lipids may remain in aqueous phase; solvent handling required	Broad range of food matrices including serum, tissue, egg yolk	Sostare et al. (36) and Matyash et al. (83)
Hexane/IPA	Hexane-isopropanol solubilizes lipids	Simple; effective for non-polar lipids	Poor recovery of phospholipids; limited polarity coverage	Neutral lipid-rich matrices (plant oils, egg yolk)	Su et al. (133) and Wan et al. (149)
Ethanol/Low-temp crystallization	Ethanol ± β-cyclodextrin precipitates proteins and extracts phospholipids	Food-grade; removes cholesterol; safe for functional foods	Moderate purity; may require multiple steps for enrichment	Dairy matrices (whey, milk, egg products)	Price et al. (28), Sprick et al. (43), Savoire et al. (60) and Haq and Chun (68)
STEP (Simultaneous Texturization and Extraction)	Combination of mechanical and solvent treatment (Ethanol + Texturization) that denatures proteins and solubilize lipids.	Single-step, scalable, efficient High PLs recovery	Requires heating, thermal and oxidation risks, process optimization needed for different matrices, potential co-extraction of protein fragments	High-moisture matrices, protein-rich by-products, egg and dairy products.	Jiménez Callejón et al. (146) and Wang et al. (88)
SHS: Switchable Hydrophilicity (Switchable solvents)	Solvents whose hydrophilicity/polarity can be rapidly changed (by CO_2_, pH, gas…) enabling selective solubilization of lipids and easy phase separation.	Sustainable method with minimal use of solvent, switchable polarity for selective extraction, solvent recovery, reduction in the purification steps.	proper solvent selection to induce this switching. Limited large-scale use in food industry.	Oily matrices and processed streams, Dairy by-products.	Rathnakumar et al. (89) and Sun et al. (90)
Natural Deep Eutectic Solvents (NADES)	Mixtures of natural components (sugars, amino acids, organic acids) form low-melting solvents	Biodegradable, tunable polarity, safe for food applications	Viscosity may slow mass transfer, poor recovery of PL	Microalgae, diary, egg yolk, marine sources	Topal et al. (31), Fouegue et al. (91) and Priharto and Dicky (92)

#### 4.1.2 Method modifications and safer solvent systems

Modifications of the classical methods have been proposed to enhance safety and reduce the number of steps (Table 2). For example, Matyash et al. (83) replaced chloroform with methyl tert-butyl ether (MTBE), generating a biphasic system where the lipid-rich layer formed on top. This made the lipids easier to collect and also increased lipid recovery, leading to an increased preference for phospholipids and neutral lipids. It has high recovery and is now the standard procedure in lipidomics, given its high compatibility with high-throughput and MS-based approaches (36, 84). Another alternative is the Hara and Radin (85) system that uses a hexane-isopropanol (3: 2 v/v) mixture, which is single-phase and non-chlorinated (Table 2). Lipid yields are comparable to the Folch method while having lower toxicity and cost.

#### 4.1.3 Soxhlet and solid-liquid extraction (SLE)

Soxhlet extraction is one of the oldest solvent-based procedures in which samples are extracted for several hours by repeated washing with a heated solvent (54, 86). Hexane, chloroform, or ethanol are commonly used as solvents. The Soxhlet method provides a fair overall lipid recovery, but it is less effective at polar lipids and often underestimates phospholipids (87). Furthermore, the long extraction times and the exposure to heat and oxygen increase the risk of oxidation and hydrolysis. For these reasons, Soxhlet is more suited for crude fat determination than for phospholipid profiling.

#### 4.1.4 Simultaneous texturization and extraction of phospholipids process (STEP)

Ethanol-Based and Hybrid Systems combining ethanol extraction with controlled heating in order to aid extraction and protein texturization have recently been developed as the Simultaneous Texturization and Extraction of Phospholipids process (88). The phospholipid recovery achieved is in the range of 70–80% from dairy or algal matrices and increases purity and processing efficiency. Furthermore, the process is a single step and shortens processing time. The process can also produce both a phospholipid concentrate and a defatted protein byproduct stream, giving it industrial advantages.

#### 4.1.5 Switchable hydrophilicity solvents (SHSs)

A new and recently developed approach is based on the use of switchable hydrophilicity solvents (SHSs). These solvents, *N*,*N*-dimethylcyclohexylamine (CyNMe₂) for example, can be reversibly switched from a hydrophobic state to a hydrophilic state upon CO₂ introduction (51, 77, 89, 90). This characteristic allows for easier extraction and selective solvent recovery without having to resort to distillation. SHSs have been able to selectively extract phospholipids in dairy or marine matrices up to ~98% with considerably less solvent.

#### 4.1.6 Natural deep eutectic solvents (NADES)

NADES are tunable, food-safe solvent systems composed of natural metabolites (sugars, organic acids, amino acids, terpenes, fatty acids) that form strong hydrogen-bond networks and lower melting points, resulting in a liquid phase at ambient conditions (Table 2) (31, 91). Hydrophobic Type V systems (menthol: carvacrol, menthol:thymol, alkanediol:fatty acid) dissolve neutral lipids (TAGs, FFAs, PUFAs), whereas hydrophilic NADES (choline chloride:glycerol or oxalic acid) can disrupt rigid matrices (92). In marine tissue, NADES systems effectively extracted neutral lipids but showed reduced efficiency for polar lipids compared with chloroform-methanol-water systems, particularly in PLs-rich matrices, indicating weaker performance for polar lipids. In fact, NADES offer advantages such as low toxicity, operation under mild conditions, tunable polarity, and potential antimicrobial or stabilizing effects, which make them attractive for food and nutraceutical use. However, their application is constrained by high viscosity that limits mass-transfer, challenges in solvent recovery and recycling, biomass-dependent extraction efficiency, and lower quantitative recovery of polar lipids (PI, LPC, CAEP) due to persistent lipid-matrix associations (31, 91).

However, the use of these methods is still limited by high cost, operation complexity, and regulatory uncertainty regarding food use. New Emergent Extraction Techniques (Green Techniques) advances in food science and technology have led to the development of new “green” extraction techniques that have more recovery efficiency of lipids (especially phospholipids), lower use of toxic solvents, and have less environmental impact (87, 93). They aim to improve yield, selectivity, and energy efficiency, and at the same time meet sustainability criteria (57, 94, 95).

### 4.2 New emergent extraction techniques (green techniques)

#### 4.2.1 Supercritical fluid extraction (SFE)

SFE employs a fluid above its critical temperature and pressure as a solvent for extracting compounds (Table 3). Carbon dioxide is the most commonly used, being an environmentally friendly solvent that can be easily removed by depressurization and does not contaminate the final product, making it suitable for producing high-purity lipid extracts. However, CO_2_ is nonpolar, limiting the solubility of phospholipids. To improve lipid extraction efficiency, CO_2_ is usually modified with ethanol, a polar cosolvent (30). Sprick et al. (43) selectively extracted phospholipids from whey protein phospholipid concentrate by using CO_2_ at 35 MPa and 40 °C in combination with 15% ethanol, achieving a final phospholipid recovery of 26.26 g PL/100 g fat, which is about two to three times higher than the recovery by conventional solvent extraction. Savoire et al. (60) applied SFE to recover phospholipids from camelina cake and scallop processing by-products using supercritical CO_2_ and ethanol. They observed total phospholipid recoveries of up to 90%. Hexadecanoic acid/steatic acid (HA/S), EPA, DHA, and PC were the predominant phospholipids present in these matrices. Phospholipid yield from salmon frame bone with supercritical CO_2_ also significantly increased when ethanol (10–20%) was added as a cosolvent (from 39 to >80%), and the extract obtained was rich in PC, PE, PS, and PI, with improved antioxidative properties (68). The SFE offers the following advantages: no solvents or solvent residues, production of high-purity extracts, no degradation of omega-3 PUFAs (EPA, DHA) sensitive to oxidation, and enhanced selectivity for polar lipid recovery. Its major limitations include the high cost of equipment and its operation, the need for careful control of extraction parameters (pressure, temperature, co-solvent addition, and extraction time), and scalability for industrial processing.

**Table 3:** Extraction yields and recovery rates of phospholipids from various matrices using different separation techniques.

Sample	Separation method	Extraction solvent/system	Qunatification/identification method	Quantity/yield of PLs	Reference
Human milk	Solid-phase microextraction (SPME)	Glass fabric @ MOF-808; eluted with DCM/MeOH (2:1, v/v) + 2% ammonia	UHPLC-QTOF-MS	Spiked recoveries for 8 PLs ranged from 76.35 to 94.25% (PC, PI, SM, LPC, PE, PS, PG, LPE)	Wang et al. (17)
Chicken liver	Enzyme-Assisted Extraction (EAE)	95% Ethanol, Protamex proteinase, 85.22 min hydrolysis	HPLC-ELSD	Phosphatidylcholine yield: 88.92% (0.89 g/mL)	Huang et al. (41)
Beta-serum (cheese plant)	SPME + Ultrasound-Assisted Extraction	Acoustic intensity 44.56 ± 3.47 W/cm^2^; CyNMe2 extraction (12:1 solvent ratio)	HPLC-CAD	Yield: 69.67 ± 3.45% MPLs	Rathnakumar et al. (42)
Dairy byproducts (buttermilk, beta-serum)	Solid Phase Extraction (SPE)	Activated silica gel + CyNMe2 (switchable hydrophilicity solvent)	UHPLC-CAD	Recovery: 98.66 ± 0.89% (buttermilk), 7.67 ± 0.51% (beta-serum)	Rathnakumar et al. (51)
Camelina press cake and scallop by-products	Supercritical Fluid Extraction (SFE)	CO₂ + ethanol (2–30%); 45 °C, 25 MPa	TLC+ Phosphorus nuclear magnetic resonance (^31^P NMR) + GC	Camelina: 84.3%, Scallop: 81.2%	Savoire et al. (60)
whey protein phospholipid concentrate (WPPC)	SFE	CO₂ + ethanol (15%), 35 MPa, 40 °C	TLC + HPLC-DAD	PLs average: 26.26 g/100 g fat	Sprick et al. (43)
whey protein phospholipid concentrate (WPPC)	Ethanol Extraction (5-stage)	70% ethanol, 70 °C	^31^P NMR	PLs content: 45.8%, Total recovery: 58.1%	Price et al. (28)
Human milk	Solid Phase Extraction (SPE)	Methacrylate base MIP	hydrophilic interaction liquid chromatography coupled with evaporative light scattering detection (HILIC-ELSD)	Recovery: 75–88%	Ten-Doménech et al. (118)
Veiled virgin olive oil (VEVO)	SPE	40 mL n-hexane with DSC-Diol or DSC-Si column	HILIC column and HPLC-ESI-qTOF-MS: HILIC with electrospray ionization quadrupole time-of-flight mass spectrometry (ESI-qTOF-MS)	Diol: 8.25 mg/kg, Silica: 4.32 mg/kg	Verardo et al. (24)

#### 4.2.2 Ultrasound-assisted extraction (UAE)

Ultrasound-assisted extraction is a novel extraction technology that has become increasingly popular as part of green chemistry solutions (96, 97). It uses acoustic cavitation to accelerate the lipid recovery process, with sound waves breaking cell walls and membranes, allowing better solvent penetration into the sample. Krishnegowda et al. (98) optimized UAE for ghee residue to achieve a higher phospholipid recovery. The phospholipid content increased from 4.98% to 35.47% of total fat, and a yield of 24.12% was achieved using 80 °C, 80% power, and 4 min as the optimal extraction parameters. Rathnakumar et al. (42) used UAE pre-treatment in combination with a liquid-liquid separation step using tertiary amines to enhance phospholipid extraction from milk and observed that phospholipid recovery increased from 7.6 to 69.7% (w/w) using this process. UAE was used to increase the extraction yield of phospholipids from microalgae (99) and fish by-products (100). UAE typically gives a higher extraction yield in a much shorter time than conventional solvent extraction methods. It also allows the use of less solvent and energy while avoiding loss of thermolabile compounds that may occur with methods using high temperatures. The process can cause oxidation of compounds, especially lipids, due to local heating. Optimization is also matrix-dependent, and larger-scale applications for industrial use are currently limited by the cost of ultrasound equipment.

#### 4.2.3 Microwave-assisted extraction (MAE)

Microwave-assisted extraction is based on the use of microwave irradiation to heat the sample matrix and the extraction solvent, thus rapidly developing the internal pressure and rupturing the cells to release the lipids of interest (101). The application of microwaves reduces the extraction time, the solvent consumption, and the thermal degradation of heat-sensitive lipids (102, 103). The optimized MAE conditions for different matrices led to high lipid recovery yields. Lipids were extracted from mozzarella with a microwave reactor at 65 °C for 15 min using ethanol as a solvent, while lipids from chicken breast yielded 2.11 g/100 g (103), which is comparable with the value obtained by Folch extraction, and the solvent used was a combination of ethyl acetate:methanol (2:1) at 54 °C. MAE extraction of lipids from fish gave a lipid recovery of 10.1 g/100 g, equivalent to the certified reference value (102), and the optimal extraction conditions in Nannochloropsis oculata microalgae were a microwave irradiation of 1 min to achieve a lipid recovery yield of 33.6% (104). MAE also helps to maintain the integrity of fatty acids and eliminates the drying step required to remove water from the sample matrix, thus decreasing the energy demand of the process. MAE is a short-time process with high efficiency, low solvent use, and potentially low environmental impact, and has advantages over other traditional lipid extraction methods from wet or complex matrices (57). It still has limitations in terms of equipment cost, uneven heating, and limited scalability for industrial applications, but its precision and environmental benefits make it a promising technology for the future.

#### 4.2.4 Enzyme-assisted extraction (EAE)

EAE employs cell-wall-degrading enzymes (cellulases, hemicellulases, pectinases, proteases) to disrupt polysaccharide-protein matrices and membrane structures, thereby releasing lipids (particularly membrane-bound phospholipids) for subsequent recovery using aqueous or mild solvents (95, 105). In comparison with conventional solvent extraction, EAE can increase efficiency and selectivity for phospholipids, reduce solvent consumption, and scale up for food applications, with primary levers being enzyme type/dose, time-temperature, and matrix properties. In a previous research, direct lipid extraction from wet Rhodosporidium toruloides showed a great extraction efficiency of 96.6% recovery via a three-step enzyme-assisted approach. The procedure included microwave pretreatment of the cells, enzymatic hydrolysis with recombinant β-1,3-glucomannanase, and solvent extraction with ethyl acetate at room temperature and atmospheric pressure (78). Likewise, Enzyme-assisted three-phase partitioning of wet Nannochloropsis gave 90.40% TFA (EA-TPP: 20% (NH_4_)_2_SO_4_, pH 6–7, 1:2 slurry:tert-butanol, 70 °C, 2 h; lipid fractions: 40.45% neutral lipids (NLs), 30.46% glycolipids (GLs), 17.79% PLs) (106). Protamex-based hydrolysis optimized PC extraction from chicken liver (PC yield 88.92%; 0.89 mg/mL) (41). Enzymatic hydrolysis of Baltic herring (0.4% Alcalase®, Neutrase®, Protamex®; 50–55 °C, 105 min) demonstrated stock-to-stock lipid variability (107). The main advantages include higher PL purity and the co-release of bioactive peptides, with clear “green” credentials (6, 95). However, there are practical challenges: enzyme cost, narrow operating windows (pH/temperature/substrate specificity), scale-up repeatability, stable emulsion formation, and potential byproducts requiring safety assessment.

#### 4.2.5 Pressurized liquid extraction (PLE)/accelerated solvent extraction (ASE)

PLE/ASE is solvent extraction conducted at elevated temperature (≈75–200 °C) and pressure (~100 atm), but still below the solvent’s critical point. Lower viscosity/surface tension and higher solubility increase mass transfer, enabling faster, fully automated, lower-solvent extractions with good reproducibility (108–111). The studies report higher total lipids and individual PLs (e.g., PI) versus conventional approaches (112). For example, Chlorella vulgaris ASE (chloroform: methanol 2:1, 100 °C, 5 min, 4 cycles) increased lipid synthesis by 6.9% (113). Isochrysis PLE (90% ethanol, 1500 psi, 80 °C; 5 min static; 3 cycles) resulted in 41.5 wt% lipids and 92.17 wt% TFA recovery (114). An optimized five-strain algal protocol used 125 °C, 3 × 3 min cycles (methanol/DMSO 9:1, then hexane/diethyl ether 1:1) and outperformed Soxhlet for low-oil strains (115). For mozzarella, ASE (MTBE/cyclohexane 30:50; 60.5 °C; 3 cycles) shifted class profiles relative to Folch: ASE favored TG, BisMePA, LPC, ZyE, LPE, ceramides; Folch favored PC, PE, PI, PS, SM. Benefits include ~90% time and ~50% solvent savings, high throughput, and broad matrix compatibility (111, 114–117). However, this procedure faces several main challenges, including the need for additional cleanup if S-PLE is not used (SPE/GPC), laborious cell packing, the use of specialized/expensive equipment, and potential thermal degradation of heat-sensitive lipids (115). Applications to food matrices remain comparatively sparse-an opportunity for further work.

### 4.3 Pretreatment and enrichment

*Solid-Phase Extraction (SPE)* is an effective technique for isolating and purifying chosen lipids, as well as enriching minor lipid classes, such as phospholipid subclasses. This is often accomplished using small cartridges (columns) filled with reversed-phase, normal-phase, or ion exchange sorbents (Table 3). These cartridges selectively retain target fractions through polar (normal phase), hydrophobic (reverse phase), or ionic interactions, allowing unwanted molecules to flow through (24, 118).

*Normal-phase silica-based chromatography for phospholipid class fractionation* has become the most immediate conduit between crude lipid extracts and high-specificity analytics for phospholipid classes (Table 4). Normal-phase silica cartridges, pre-conditioned with CHCl_3_:MeOH (95:5, v/v), have been reported to reproducibly deplete neutral lipids, followed by elution of PLs with MeOH and CHCl_3_:MeOH:H_2_O (5:3:2, v/v/v). Across multiple dairy streams, this workflow has been shown to produce high-purity milk PLs (MPL) fractions while resolving shifts in class composition introduced by upstream solvent chemistries (e.g., CyNMe2 vs. Folch), often with selective enrichment of PI and PE (51, 77, 89). Similar silica-based schemes have also been implemented for marine and fish oils when class-directed cleanup is needed before quantification (107). In short, Silica-based normal-phase provides reproducible class isolation, lowers chromatographic matrix effects, and standardizes inputs for downstream HPLC/LC–MS and ^31^P NMR (87).

**Table 4:** Representative analytical workflows for phospholipid extraction, separation, and quantification across food matrices.

Matrix (food/product studied)	Extraction method	Separation/quantification/identification method	Main outputs/results	Advantages	Disadvantages	Reference
Dairy wastes (beta-serum)	switchable solvents (CyNMe₂)	Solid-Phase Extraction (SPE) UHPLC-CAD	Heighst PLs extraction yield was 79.39%. PI (~41%), PE (~34%), PS (~6%), PC (~9%), SM (~8%)	High purity recovery, scalable for dairy waste valorization	Solvent-intensive, multiple steps	Rathnakumar et al. (89)
Buttermilk (BM) and Buttermilk serum (BS)	switchable solvents (CyNMe₂) vs. Folch	Solid-Phase Extraction (SPE) UHPLC-CAD	CyNMe₂ yielded higher MPL conc. BM: 1897.5 μg/mL; BS: 2797.7 CyNMe₂ BM: PI (37.9%), PC (28.6%). CyNMe₂ BS: PI (34.3%), PE (17.3%)	CyNMe₂ selective for PI, higher enrichment	CyNMe₂ may alter proteins	Rathnakumar et al. (51)
Raw cream (RC), Buttermilk (BM), Concentrated BM (CBM), Beta-serum (BS)	Switchable solvent extraction vs. Folch	Solid-phase extraction (SPE) + Thin-Layer Chromatography (TLC)	PL recovery improved by SHS; BM recovery up to 99.9%; CBM recovery up to 77.3%; BS recovery 7.6% Bands detected for PE, PS, PI, PC, SM. SHS-extracted PLs showed more intense bands vs. Folch/ME	High recovery with SHS; good PLs separation with SPE	Matrix-dependent efficiency, laborious	Cheng et al. (77)
Baltic herring oil	Enzyme assisted aqueous extraction	Separation by SPE (Sep-Pak Silica) GC-FID for Fatty acids NF and PF were separated and detected using UHPLC–MS equipped with an ESI source Identification of PLs was aided by using a Cortecs UPLC HILIC	PLs yields of 0.01–0.02% oil sample. PUFA-rich oil: MUFA+PUFA 75%; n-3 PUFAs 27% (EPA 6.2%, DHA 11.5%) 9.5% PLs detected (PC 60%, PE 19.2%, PI 11.2%, SM 0.3%); TAGs 42.8%	Effective class fractionation, reproducible PUFA profiling Comprehensive lipidomics, molecular species resolution	Low-fat yield, limited PLs compared to salmon Expensive instrumentation	Aitta et al. (107)
Egg yolk and Soybean	Lipid extraction + TLC Tailored Convinient Solvent extraction using chloroform, ethanol, and acetone as solvents for egg yolk Followed by ethanol-based refluxing for Soybean	TLC on silica plates, solvent tailored (ethyl acetate/methanol 3:2 for EY; 2-propanol/methanol 3:1 for soybean) (a) High-performance liquid chromatography (HPLC)/ liquid chromatography-mass spectrometry (LC–MS) analysis	PL classes identified (PC, PE, minor). PC recovery ~7.5% (EY) and 6.4% (soybean) EPC and SPC exhibited purities of 91.37 and 82.52%, respectively LC–MS validated the molecular composition of the purified PC (m/z values around 780.6685 and 780.6639)	Simple, cost-effective, adaptable solvent systems	Poor sensitivity/resolution, semi-quantitative	Hajimirzaei et al. (119)
Microalgae	Enzyme-assisted -three-phase partitioning (EA-TPP extraction)	Silica gel column chromatography GC-FID after FAME derivatization	Lipid yield 88.7% after 2 cycles; composition: NLs 40.5%, GLs 30.5%, PLs 17.8%	High efficiency, separates lipid classes	Multi-step, requires FAME derivatization	Qiu et al. (106)
Chicken liver	Solvent extraction + TLC + HPLC-ELSD	TLC (CHCl₃: MeOH: H₂O 65:25:4) + HPLC-ELSD quantification	PC identified as main PL (Rf = 0.78). HPLC-ELSD quantified PC & PE; PCCL concentration = 0.89 mg/mL	Combined TLC + HPLC improves reliability, good quantification	Requires standards, solvent heavy	Huang et al. (41)
Mozzarella cheese	ASE extraction	UHPLC-Q-Orbitrap-MS	Identified 13 lipid subclasses, including PCs, SMs, LPCs, PEs, PIs	High resolution, untargeted profiling, structural info	Costly, complex instrumentation	Mentana et al. (111)
Soft cheese	MAE extraction	UHPLC-Q-Orbitrap-MS + LipidSearch database	Identified oxidized GPLs; TGs most abundant; SFA-rich sn-1 position	Enables oxidized lipid identification, detailed FA distribution	Requires proprietary software, high expertise	Campaniello et al. (128)
Chicken breast	MAE extraction	FAMEs + GC-FID (P-SIL 88 column)	Major FAs retained: oleic (627 mg/100 g), linoleic (459), palmitic (415), stearic (113)	High sensitivity and accuracy for FA profile	Cannot analyze intact PLs, derivatization needed	Medina et al. (103)
Egg yolk and enzymatically modified egg yolk	Conventional Folch with Repeated methanol extractions	Phosphorus-31 nuclear magnetic resonance spectroscopy (^31^P NMR)	The solubilized (lyso)phospholipids recoveries ranging between 96 and 108%. Egg yolk mostly contained phosphatidylcholine (PC, 61.6 mg/g) and phosphatidylethanolamine (PE, 15.9 mg/g), with low levels of sphingomyelin (SM, 1.5 mg/g), phosphatidylinositol (PI, 1.4 mg/g), and traces of lysophospholipids. Enzymatically modified egg yolk was largely composed of lysophosphatidylcholine (1-LPC, 37.0 mg/g; 2-LPC, 2.9 mg/g) and lysophosphatidylethanolamine (1-LPE, 8.7 mg/g; 2-LPE, 1.1 mg/g), with only trace amounts of residual PC and PE remaining	Does not need derivatization Delivers accurate profiling; it has shorter relaxation times that enhance measuring speed while maintaining precision. Valuable research technique for thorough phospholipid investigation.	Poor sensitivity, requires greater sample volumes, and the high cost of instrumentation.	Mayar et al. (132)
Egg yolk	Ethanol; then 75 mL of hexane Cold acetone was used to precipitate and wash egg yolk phospholipids	Phosphorus-31 nuclear magnetic resonance spectroscopy (^31^P NMR)	PC dominance (104 μmol/100 mg, 77%), followed by PE (24.4 μmol/100 mg, 18%), while SM and LPC were minor (3.57 μmol/100 mg each, ~3%)			Zhao et al. (37) and Zhao et al. (38)

GC-FID, gas chromatography coupled with flame ionization detection; UHPLC–MS, ultra-high performance lipid chromatography coupled with triple quadrupole mass spectrometer; UHPLC, HPLC coupled with a charged aerosol detector (CAD); UHPLC-Q-Orbitrap-MS, UHPLC coupled to a Q-Exactive Focus Orbitrap Mass Spectrometer equipped with a heated electrospray ionization (HESI) source.

## 5 Analytical methods for phospholipid identification and quantification in food matrices

After obtaining the phospholipids from the dietary samples, the next significant step is to separate, identify, and quantify them. In food matrices, these steps are quite difficult due to the amphiphilic nature, structural variety, and the requirement to discover small components of phospholipids within complex lipid mixtures. To overcome these problems, a variety of analytical techniques have been developed and are frequently used in tandem, depending on the target PL classes, sample type, and desired sensitivity. Over the years, methodologies have evolved from simple colorimetric tests and thin-layer chromatography (TLC) to more sophisticated techniques like UHPLC, HPLC–MS, and multidimensional lipidomics.

In this section, we tried to summarize the key analytical techniques employed in the separation, identification, quantification, and structural characterization of phospholipids, highlighting the recent advancements that have significantly enhanced sensitivity, resolution, and lipidome coverage. Table 3 presents a comparative summary of different analytical methodologies.

### 5.1 Class-level screening and quantification

*Thin-layer chromatography (TLC)* remains a quick, low-cost screen for class presence/absence and process verification. Matrix-tuned mobile phases on silica (or cellulose) separate major PL classes; visualization with molybdophosphoric acid/iodine enables qualitative or semi-quantitative readouts (Table 3). In food PLs analysis, TLC is frequently used to determine the PLs classes in egg yolk and soybean lipid extracts, which include PC, PE, and minor components (119). An optimal ethyl acetate/methanol (3:2 v/v) solvent system was utilized for egg yolk, whereas 2-propanol/methanol (3:1 v/v) was used for soybean, indicating the necessity to tailor the solvent system to the chemical composition of the raw material. TLC demonstrated that the purification procedure effectively eliminated contaminants, resulting in fewer spots and high PC recovery rates (7.45% for egg yolk and 6.37% for soybean). This multistep technique emphasizes the importance of solvent polarity and specificity in selectively enriching phospholipids while remaining efficient. TLC is well-suited for confirming enrichment (e.g., PC/PE in egg/soy) and for visual checks after unconventional extraction chemistries (6, 77, 119, 120). But its modest resolution and laborious quantitation preclude sole reliance on rigorous compositional studies.

*UPLC/HPLC with Universal Detectors or MS*: For class-level quantification in foods, normal-phase HPLC/UHPLC coupled to ELSD or CAD is the current workhorse (Table 3). Silica columns with appropriately buffered ACN/MeOH gradients resolve PI, PE, PS, PC, and SM with robust response factors and throughput (46, 56, 121). In dairy MPLs, CAD methods have been applied to capture extraction-parameter effects (time, temperature, solvent ratio) on class distributions and have documented substantial MPL concentration after CyNMe2 workflows (51, 89). Analyzing milk phospholipids (MPLs) using UHPLC coupled with a charge aerosol detector (CAD), five primary phospholipid classes: PI, PE, PS, PC, and SM, were identified after separation on two silica columns (89). The extraction conditions strongly influenced the PLs class distribution: prolonged extraction (18 h, 60 °C, solvent ratio 1:18) resulted in high total MPLs recovery (79.39 ± 5.55%) dominated by PI (41.77%) and PE (34.59%), whereas shorter extraction (3 h, 60 °C, 1:3) resulted in lower recovery (10%) with enrichment of SM (39.68%) and PC (28.07%), highlighting the sensitivity of class-level profiles to upstream processing parameters. Similarly, individual buttermilk and butter serum phospholipids were quantified using the same system (UHPLC system coupled with CAD) following a solid-phase extraction. Comparative analysis showed that extraction using CyNMe2 significantly enhanced recovery of acidic phospholipids, particularly PI and PE, compared to the conventional Folch method, while also improving SM recovery in butter milk. Targeted HPLC-ELSD [Waters 2998 system with an Intersil SIL-100A column (150 mm × 4.6 mm, 5 μm)] has also been used to validate PC-rich fractions from non-dairy tissues, chicken liver phosphatidylcholine (PCCL) (41). The HPLC-ELSD analysis revealed that PCCL included PC and PE, with retention durations of 18.6 min and 8.5 min, respectively. Using a calibration curve with highly pure PC (≥98%), the concentration of PCCL was found to be 0.89 mg/mL, with peak shapes and retention periods matching the standard, validating the method’s appropriateness for quantitative analysis and comparable purity to previously reported values. These studies showcase how UHPLC/HPLC, coupled with universal detectors, enables robust, reproducible quantification of major PLs classes and provides insight into the extraction-dependent variabilities in PLs composition across complex dairy matrices. However, among the drawbacks of this system are the co-elution risks for minor Lyso-species and the need for class standards for accurate response normalization (Tables 3, 4).

*Enzymatic and Colorimetric Assays* targeting choline or phosphate (via PLD or coupled enzyme systems) provide rapid, accessible readouts for total PLs and selected classes, supporting screening and high-throughput studies where instrument time is constrained (Table 4) (122–125). While newer fluorometric panels broaden class coverage (PC, PE, PS, PA, PI, PG + CL, SM), specificity remains lower than LC-MS or ^31^P NMR; thus, these assays are best positioned as complementary quantifiers rather than stand-alone structural tools (123, 124).

### 5.2 Molecular-level and compositional analysis

*Gas chromatography of fatty acid methyl esters (GC-FAME)* complements class analytics by resolving acyl distributions of separated PL fractions or totals (Table 4). While GC cannot profile intact PLs, it offers high precision for acyl signatures, enabling functional inference (e.g., higher PUFA content in PE vs. PC) and cross-method validation; MAE-derived lipidomes have shown parity with Folch by GC-FID benchmarks (10, 99, 126). For example, lipids extracted by MAE from chicken breast samples were transformed into fatty acid methyl esters (FAMEs) (103). To evaluate their fatty acid profile, these FAMEs were separated on a highly polar capillary column (P-SIL 88 column) using gas chromatography (GC) fitted with a flame ionization detector and split injector (GC-FID). The resultant fatty acid profile was quite similar to that obtained by Folch extraction. Major fatty acids, such as oleic, linoleic, palmitic, and stearic acids, were retained. These findings show that GC-FAME can provide robust and reproducible fatty-acyl profiling data that are largely independent of the used extraction method. However, due to the derivatization and the loss of the intact PLs molecules, GC-based approaches are not suitable for direct PLs class or molecular-species analysis. Instead, GC-FAME serves as a high-precision complementary technique for validating fatty acid composition in PLs fractions and supporting functional or nutritional interpretation. In summary, we can say that GC is not suited for direct analysis of intact phospholipids, although it is commonly used to assess the fatty acid profiles of separated phospholipid fractions. Its key strength is its accuracy and sensitivity for fatty acid identification, but one of its limitations is its inability to offer structural information on intact phospholipid molecules.

*LC-ESI-MS(/MS)* has become indispensable for structural resolution at the molecular-species level (Table 4). Positive-mode ESI favors choline lipids (PC, SM); negative mode captures acidic PLs (PE, PI, PS, PA). MS/MS provides headgroup-diagnostic ions and acyl assignments, enabling species-resolved quantification, oxidation tracking, and Lyso-lipid detection (44, 47, 121, 127). Applications span comprehensive food lipidomics, e.g., UHPLC–MS profiling of fish oils and cheese extracts (107, 111, 128), and QC contexts such as infant-formula PL mimicry (64, 126). For instance, UHPLC–MS analysis of Baltic herring lipids identified and quantified multiple PLs classes such as PC, PE, PI, PG, LPC, LPE, and SM, with dominance of PC (60%), followed by PE and PI, showing the suitability of MS with the comprehensive PLs profiling in marine matrices (107). An untargeted lipidomics of dairy products using UHPLC-Q-Orbitrap-MS allowed the identification of numerous lipid subclasses in both positive and negative ion mode, including PLs, lysophospholipids, and sphingolipids (111). MS provides critical insight into the structural diversity of individual PLs classes. Detailed molecular-species and sn-position analysis of PC revealed distinct fatty acyl distributions and the presence of oxidized species, underscoring the unique capability of MS-based platforms to resolve structural features at a level unachievable by chromatographic detection alone (128). The approach is analytically dominant but benefits from clean inputs (SPE or class-directed LC) to curb ion suppression and isobaric interferences. While MS offers incomparable molecular-species-level resolution, complementary spectroscopic techniques such as ^31^PNMR provide independent, quantitative validation of PLs class composition, as discussed in the following section.

*Nuclear Magnetic Resonance (^31^PNMR)* offers non-destructive, inherently quantitative class profiling of phosphorus-containing lipids, making it a strong orthogonal reference to chromatographic and MS methods (Table 4) (58, 129, 130, 143). ^31^PNMR signal intensities are directly proportional to molar concentration, which enables reliable class-level quantification of PLs such as PC, PE, PI, PS, and SM without the need for external calibration standards. Among the significant applications include the profiling of egg yolk, krill oil, soybean lecithin, and dairy products, where PC and PE were consistently identified as dominant classes, and enzymatically modified samples showed marked conversion to lysophospholipid species (37, 38, 131, 132). The reported recoveries and repeatability support routine deployment for lipid-rich matrices, illustrating their specificity and utility in validation workflows. The main limitation is the lack of sensitivity and capital cost.

## 6 Synthesis and method selection

For PLs research and quality control in foods, an effective, and widely applicable analytical framework is: (i) extraction adapted to the matrix of interest; (ii) SPE to remove neutral lipids and pre-enrich PLs; (iii) UHPLC-CAD/ELSD for routine class-level quantification; (iv) LC–MS(/MS) for species-level resolution and tracking of oxidation/Lysophospholipid formation; (v) ^31^P NMR for orthogonal class quantification and method validation; and (vi) GC-FAME for acyl fingerprinting.

This integrated, multi-tiered approach offers analytical depth to be adapted/adjusted according to matrix complexity and study objectives, balancing throughput, specificity, and reproducibility across various food matrices. It also makes the effects of upstream solvent choice on the overall class distribution explicit, facilitating direct comparisons.

## 7 Conclusion and future prospects

Classical solvent-based extraction methods (e.g., Folch and Bligh and Dyer) are still the most frequently used procedures for PLs extraction due to their simplicity and good recoveries. However, the use of toxic organic solvents is becoming an issue in the food and environmental sectors in the search for new “greener” methodologies that minimize the use of hazardous solvents and energy. The recently developed green technologies (ultrasound-, enzyme-, and supercritical fluid–assisted extractions) are characterized by increased selectivity, reduction in solvent use, and higher compatibility with the possible thermal degradation or oxidation of heat- and oxidation-sensitive PLs. These techniques will be further optimized, leading to improved extraction yields with minimal environmental impact and in favor of sustainable production of high-value food, nutraceutical, and pharmaceutical products. The implementation of modern and high-resolution analytical platforms such as UHPLC-CAD, LC–MS/MS, MALDI-TOF, and ^31^P NMR has recently made it possible to precisely profile lipids in a high-throughput mode. However, the lack of reproducibility among these analyses due to the variability in extraction and quantification procedures is a barrier that will have to be overcome in the future. This will require further standardization, automation, and real-time monitoring of the extraction process, which will facilitate comparison between studies. In all cases, the best extraction strategy will depend on the main goal to be achieved (high extraction yield, keeping the native structure, using the lowest amount of energy, or applying a sustainable strategy). Overall, the present paper emphasizes that the selection of PLs extraction and analytical strategies must remain matrix-aware and objective-driven, balancing recovery, molecular integrity, energy efficiency, and sustainability rather than relying on a single universal protocol.
